# Diversity and Physiological Characterization of D-Xylose-Fermenting Yeasts Isolated from the Brazilian Amazonian Forest

**DOI:** 10.1371/journal.pone.0043135

**Published:** 2012-08-13

**Authors:** Raquel M. Cadete, Monaliza A. Melo, Kelly J. Dussán, Rita C. L. B. Rodrigues, Silvio S. Silva, Jerri E. Zilli, Marcos J. S. Vital, Fátima C. O. Gomes, Marc-André Lachance, Carlos A. Rosa

**Affiliations:** 1 Departamento de Microbiologia, Instituto de Ciências Biológicas, Universidade Federal de Minas Gerais, Belo Horizonte, Brazil; 2 Departamento de Biotecnologia, Escola de Engenharia de Lorena, Universidade de São Paulo, São Paulo, Brazil; 3 Embrapa Agrobiologia, Seropédica, Rio de Janeiro, Brazil; 4 Departamento de Biologia, Universidade Federal de Roraima, Campus do Paricarana, Boa Vista, Brazil; 5 Departamento de Química, Centro Federal de Educação Tecnológica de Minas Gerais, Belo Horizonte, Brazil; 6 Department of Biology, University of Western Ontario, London, Ontario, Canada; University of Wisconsin, Food Research Institute, United States of America

## Abstract

**Background:**

This study is the first to investigate the Brazilian Amazonian Forest to identify new D-xylose-fermenting yeasts that might potentially be used in the production of ethanol from sugarcane bagasse hemicellulosic hydrolysates.

**Methodology/Principal Findings:**

A total of 224 yeast strains were isolated from rotting wood samples collected in two Amazonian forest reserve sites. These samples were cultured in yeast nitrogen base (YNB)-D-xylose or YNB-xylan media. *Candida tropicalis, Asterotremella humicola, Candida boidinii* and *Debaryomyces hansenii* were the most frequently isolated yeasts. Among D-xylose-fermenting yeasts, six strains of *Spathaspora passalidarum*, two of *Scheffersomyces stipitis*, and representatives of five new species were identified. The new species included *Candida amazonensis* of the *Scheffersomyces* clade and *Spathaspora* sp. 1, *Spathaspora* sp. 2, *Spathaspora* sp. 3, and *Candida* sp. 1 of the *Spathaspora* clade. In fermentation assays using D-xylose (50 g/L) culture medium, *S. passalidarum* strains showed the highest ethanol yields (0.31 g/g to 0.37 g/g) and productivities (0.62 g/L·h to 0.75 g/L·h). *Candida amazonensis* exhibited a virtually complete D-xylose consumption and the highest xylitol yields (0.55 g/g to 0.59 g/g), with concentrations up to 25.2 g/L. The new *Spathaspora* species produced ethanol and/or xylitol in different concentrations as the main fermentation products. In sugarcane bagasse hemicellulosic fermentation assays, *S. stipitis* UFMG-XMD-15.2 generated the highest ethanol yield (0.34 g/g) and productivity (0.2 g/L·h), while the new species *Spathaspora* sp. 1 UFMG-XMD-16.2 and *Spathaspora* sp. 2 UFMG-XMD-23.2 were very good xylitol producers.

**Conclusions/Significance:**

This study demonstrates the promise of using new D-xylose-fermenting yeast strains from the Brazilian Amazonian Forest for ethanol or xylitol production from sugarcane bagasse hemicellulosic hydrolysates.

## Introduction

Growing environmental concerns over the use and depletion of non-renewable fuel sources, together with the rising price of oil and the instability of the oil market, have stimulated interest in optimizing fermentation processes for the large-scale production of alternative fuels such as ethanol [1]. The largest potential feedstock for ethanol is lignocellulosic biomass, which includes materials such as agricultural residues (corn stover, crop straws, sugarcane bagasse), herbaceous crops, short rotation woody crops, forestry residues, waste paper and other plant wastes [2].

Lignocellulosic biomass varies among plant species but generally consists of ∼25% lignin and ∼75% carbohydrate polymers (cellulose and hemicellulose). It is the largest known renewable carbohydrate source. The cellulosic and hemicellulosic portions of biomass can be separated from the lignin and depolymerized by hydrolysis to obtain their constituent sugars, mainly glucose from cellulose and D-xylose from hemicellulose [3]. As the major sugar in hemicellulose, D-xylose is the second most abundant sugar in lignocellulose [4]. The successful conversion of hemicellulose into fuel ethanol at high yields is the deciding factor for the economic viability of the process [5]. Thus, the efficient use of lignocellulosic biomass as a substrate for ethanol production requires effective utilization of D-xylose [4].

Yeasts that produce ethanol from D-xylose have been isolated from various locations, including tree exudates [6], wood-boring insects [7], [8], decaying wood [7], [9], rotten fruit and tree bark [10]. Known D-xylose-fermenting yeasts are principally from the species *Scheffersomyces (Pichia) stipitis, Candida shehatae, C. lignosa, C. insectosa, C. tenuis, Pachysolen tannophilus* [11]–[13], *Spathaspora passalidarum* [14] and *S. arborariae* [9], [15]. Among these naturally D-xylose-fermenting yeasts, *S. stipitis* and *S. passalidarum* are considered the best ethanol producers [11], [16]. Despite the existence of these microorganisms, it is still challenging to reach high yields of ethanol from pentose sugars on a large scale [17] because no microorganisms that robustly convert pentose sugars into ethanol at high yields while withstanding fermentation inhibitors have been identified [18].

According to Jeffries and Kurtzman [19], the identification of yeast strains that ferment hemicellulosic sugars will improve prospects for lignocellulosic ethanol production. The strains can be obtained by isolating them from the environment, by mutating and selecting strains in the laboratory [20] or by engineering strains of *Saccharomyces cerevisiae* that can ferment pentoses [21].

The Amazon basin sustains almost 60% of the world’s remaining tropical rainforest, with Brazilian Amazonia alone comprising ∼30% of the world’s current primary tropical rainforests. This environment plays crucial roles in biodiversity conservation, carbon storage, and regional hydrology and climate [22], [23]. Even considering all of the research that has been performed on biodiversity in the Amazonia to date, clearly much more research is needed to understand the enormous diversity and complexity of this region. Few studies have characterized the yeast diversity of Brazilian Amazonian environments [24]–[26]. Works related to yeast diversity in the region have identified a number of potential new species, but only one species, *Candida amapae* (*Saccharomycopsis* clade), from the region was characterized [25]. In this work, we studied yeast diversity in rotting wood collected from two Amazonian sites, focusing on the isolation of new D-xylose-fermenting yeasts that might potentially be used in the production of ethanol from sugarcane bagasse hemicellulosic hydrolysates.

## Materials and Methods

### Yeast Isolation

Yeasts were isolated from rotting wood samples collected in two sites of Amazonian Forest in the state of Roraima, in northern Brazil. These sites are maintained by Embrapa (Empresa Brasileira de Pesquisa Agropecuária)-Roraima for long-term experiments and are located in the municipalities of Mucajaí (2° 25′ 48′′N and 60° 55′ 11′′W) and São João da Baliza (00° 56′ 58′′N and 59° 54′ 41′′W). The collection sites were the experimental ecological reserve Serra da Prata (Mucajaí), belonging to Embrapa-Roraima, and the ecological reserves belonging to the owners of private landOsvaldo Antônio Sant’ana (Mucajaí) and José Lopes (São João da Baliza). All necessary permits were obtained from Embrapa-Roraima (collection permission obtained by Jerri E. Zilli) and from the owners of the private lands for the described field studies. The predominant vegetation in these sites is characterized as an Amazonian Forest biome. The climate is hot and humid, with an annual precipitation between 1,300 and 2,900 mm, and the average temperature ranges from 25.6 to 27.6°C. The Amazonian Forest comprises a *continuum* of nine main, floristically distinct vegetal formations, and 70% of it is occupied by upland forests that are characterized by their high richness and diversity of tree species [27]. The field collections were made according to the Brazilian diversity rules.

Forty decayed wood samples, 20 samples from each site, were collected in October 2009. Each sample was collected approximately 5 m from the other. The samples were stored in sterile plastic bags and transported under refrigeration to the laboratory within 24 h. One gram of each sample was placed, separately, in 125 mL Erlenmeyer flasks with 20 mL of sterile YNB-D-xylose medium (yeast nitrogen base, 6.7 g/L; D-xylose, 5 g/L; chloramphenicol, 0.2 g/L) or 20 mL sterile YNB-xylan medium (yeast nitrogen base, 6.7 g/L; xylan, 10 g/L; chloramphenicol. 0.2 g/L; pH 5.0±0.2). D-xylose, xylan and YNB solutions were sterilized separately. The flasks were incubated at 25°C on an orbital shaker at 150 rpm for 3–10 days. When growth was detected, 0.5 mL was transferred to a tube containing 5 mL sterile YNB-D-xylose or YNB-xylan media. The tubes were incubated on an orbital shaker as described above. After growth detection, one loopful of each tube was streaked on YNB-D-xylose or YNB-xylan agar media. The plates were incubated at 25°C until yeast colonies developed [9]. The different yeast morphotypes were purified by restreaking on yeast extract–malt extract agar (YMA – glucose, 10 g/L; peptone, 5 g/L; yeast extract, 3 g/L; malt extract, 3 g/L; agar, 20 g/L) and stored at −80°C or in liquid nitrogen for later identification.

### Yeast Identification

The yeasts were preliminarily grouped according to various characteristics including their colony morphology and standard tests for growth on different carbon and nitrogen sources [28]. Physiology-based groupings were confirmed by PCR fingerprinting using the Intron Splice Site primer EI-1 (5′CTGGCTTGGTGTATGT) [29]. Yeast strains with identical DNA banding patterns were grouped and putatively considered to belong to the same species [30]. At least one representative strain from each EI-1 PCR group was subjected to sequence analysis of the D1/D2 region and internal transcribed spacer (ITS) domains of the large subunit of the rRNA gene as described below. Physiologically distinct strains with unique EI-1 PCR banding patterns were also selected for direct identification by sequencing of the D1/D2 region and ITS domains.

The D1/D2 and ITS domains were amplified by PCR directly from whole cells as described previously [31]. The amplified DNA was concentrated, cleaned and sequenced in a Mega-BACE™ 1000 automated sequencing system (Amersham Biosciences, USA). Potential new species were also sequenced using an ABI sequencer at the John P. Robarts Research Institute, London, Ontario, in Canada. The sequences were assembled, edited and aligned with the program MEGA5 [32]. Sequences of the new species isolated in this work were deposited in GenBank. The existing sequences for other yeasts were retrieved from GenBank. Phylogenetic placements of new species were based on maximum parsimony analysis of the sequences of the D1/D2 domains of the large subunit of the rRNA gene.

### Screening of D-xylose-fermenting Yeasts

The ability to ferment D-xylose was tested in Durham tubes containing a 2% (w/v) solution of the sugar. The tubes were incubated at 25°C on an orbital shaker at 100 rpm for 25 days and observed daily for gas production. *Candida shehatae* CBS 5813, *C. insectosa* CBS 4286, *C. lignosa* CBS 4705 and *S. stipitis* NRRL Y-7124 were used as positive controls for D-xylose fermentation [9].

Yeasts showing the development of gas inside the Durham tubes were tested for fermentation in D-xylose culture medium (YPX: yeast extract-peptone-D-xylose medium) as described below. In addition, the yeast isolates identified by D1/D2 rRNA gene sequencing as belonging to the D-xylose-fermenting clades *Spathaspora* or *Scheffersomyces* were also tested for fermentation in YPX medium. Yeasts with the best D-xylose consumption and highest ethanol yields (Y_p/s_^et^) were assayed for their ability to ferment sugars in sugarcane bagasse hemicellulosic hydrolysate. *Candida lignosa* CBS 4705 and *S. stipitis* NRRL Y-7124 were used as positive controls.

### Fermentation Assays

#### Inoculum preparation

Yeast inocula were prepared on yeast extract–malt extract agar (YMA – glucose, 10 g/L; peptone, 5 g/L; yeast extract, 3 g/L; malt extract, 3 g/L; agar 20 g/L) plates at 30°C for 24–48 h. Cells were cultured in 50 mL YPX liquid medium (yeast extract, 10 g/L; peptone, 20 g/L; D-xylose, 30 g/L) in 125 mL Erlenmeyer flasks at 30°C with continuous shaking (200 rpm) for 24 h at 30°C. D-xylose and yeast extract-peptone solutions were sterilized separately. Cells were recovered by centrifugation at 2,600×*g* for 20 min, washed twice and resuspended in the fermentation media to a final concentration of 0.5 g/L.

#### Medium composition and cultivation conditions

Batch fermentation experiments were carried out in 50 mL D-xylose culture medium (yeast extract, 10 g/L; peptone, 20 g/L; D-xylose, 50 g/L), pH 6.0, in 125 mL Erlenmeyer flasks incubated as described above for 48 h. Fermentation was monitored by taking samples at 0, 12, 24 and 48 h.

Sugarcane bagasse was supplied by Usina São Francisco (Sertãozinho, SP, Brazil). Hemicellulosic hydrolysate was prepared as described previously [33] in a 250 L stainless steel reactor loaded with sugarcane bagasse and sulfuric acid solution (100 mg acid/g dry matter). The reactor was operated with a solid/liquid ratio of 1∶10 at 121°C for 20 min. After hydrolysis, the resulting solid material was removed by filtration. The hemicellulosic hydrolysate was concentrated in a 30 L evaporator at 70±5°C to obtain a xylose concentration of about 70 g/L. To reduce inhibitors, a detoxification assay was performed as described in Alves et al. [34] by first raising the pH to 7.0 with calcium oxide and then decreasing it to pH 5.5 with phosphoric acid, adding active charcoal (2.5% w/v) and incubating at 200 rpm at 30°C for 1 h. The precipitates resulting after each procedure were removed by vacuum filtration. The sugar composition of the hydrolysate before autoclaving was 59 g/L D-xylose, 6.2 g/L glucose and 6.4 g/L L-arabinose. The treated hydrolysate was autoclaved at 111°C for 15 min and supplemented with yeast extract solution (3 g/L). Experiments were carried out in 250 mL flasks with 100 mL supplemented hemicellulosic hydrolysate. The average hydrolysate was at pH 5.1 and composed of 50.2 g/L D-xylose, 5.3 g/L glucose, 5.8 g/L L-arabinose, 3.2 g/L acetic acid, 0.01 g/L furfural, 0.01 g/L hydroxymethylfurfural, and 1.3 g/L total phenols. The flasks were incubated as described above for 96 h. Samples were taken at 0, 12, 24, 48, 72 and 96 h. Samples were stored at −20°C until analysis. All experiments were performed in duplicate.

### Analytical Methods

Cell concentrations were determined by correlating optical density (OD) measurements taken with a Beckman DU 640B spectrophotometer at 600 nm with a previously constructed calibration curve (dry weight × optical density). After the cell concentration determined, samples were centrifuged at 2,600×*g* for 15 min, and the supernatant was diluted and filtered using a Sep-Pak C18 (Millipore) filter. Monosaccharides (glucose, D-xylose and L-arabinose), xylitol, glycerol, ethanol, and acetic acid levels were determined by HPLC (Waters 410, Milford, MA, USA) using a Bio-Rad Aminex HPX-87H (300×7.8 mm) column at 45°C with a sample injection volume of 20 µL, a Waters 410 refraction index, a mobile phase of 0.01 N H_2_SO_4_ and a flow rate of 0.6 mL/min.

### Fermentation Parameter Calculation

The fermentation parameters Y_p/s_^et^ (g/g, ethanol yield), Y_p/s_^xy^ (g/g, xylitol yield), Qp (g/L·h, ethanol productivity), η (%, fermentation efficiency) and D-xylose and/or glucose consumption (%) were experimentally determined. Ethanol (Y_p/s_^et^, g/g) and xylitol (Y_p/s_^xy^, g/g) yields were calculated following the methods of Schmidell et al. [35]), which correlated ΔP produced ( ΔP_ethanol_ or ΔP_xylitol_) with ΔS consumed (substrate consumed to product obtained, derived by determining the total, initial and consumed substrate). The slope of the line through the origin provided the estimate of Y_p/s_^et^ and Y_p/s_^xy^. Ethanol productivity (Qp, g/L·h) was determined by the ratio of ethanol concentration (g/L) to fermentation time (h). Conversion efficiency (η, %) was calculated as a percentage of the maximum theoretical ethanol yield (0.51 g ethanol/g D-xylose and/or glucose). D-xylose and/or glucose consumption (%) was determined as a percentage of the initial sugar concentration.

## Results and Discussion

### Yeast Isolation and Diversity

In this work, we studied the diversity of rotting-wood-associated yeasts from two Amazonian sites. A total of 224 yeast strains were isolated from rotting wood samples from the forest reserve sites of São João da Baliza (114 yeast strains) and Mucajaí (110 yeast strains). Of these strains, 118 were obtained following growth in YNB-D-xylose medium, and 106 were obtained from growth in YNB-xylan medium.

Table 1 shows the results of yeast species identification, the occurrence of each species by isolation site, the number of samples cultured in both media (YNB-D-xylose and xylan) and the results of the Durham tube fermentation tests. Of the 33 yeast species identified, 26 species were previously known and seven were new (Table S1). Eleven species were isolated from both isolation sites, whereas 22 species were observed in only one site. Sixteen of the 22 species were observed in the São João da Baliza forest reserve and the remaining six were observed in the Mucajaí forest reserve. Species in the genus *Candida* (namely 16 species related to the *Candida glaebosa*, *Kurtzmaniella, Lindnera, Lodderomyces*/*Spathaspora, Metschnikowia*, *Ogataea*, *Wickerhamomyces* and *Yamadazyma* clades were the most prevalent, followed by the genus *Spathaspora*, with four species. *Candida tropicalis* (*Lodderomyces*/*Spathaspora* clade) was the most frequently isolated yeast, occurring in 15 samples cultured on YNB-D-xylose medium and 13 samples in YNB-xylan medium, followed by *Asterotremella humicola* (eight samples on YNB-D-xylose medium and 10 in YNB-xylan medium) and *Candida boidinii* (*Ogataea* clade; 10 samples on YNB-D-xylose medium and seven samples on YNB-xylan medium). Strains of *C. tropicalis* have been reported in fruit, flowers, soil, water, and clinical specimens [36], and this species has already been shown to produce ethanol and, mainly, xylitol from D-xylose [37]–[40]. Strains belonging to the genus *Asterotremella,* including *A. humicola*, have been isolated from soil, plants and mushrooms [41]. *Candida boidinii* has been found with high regularity in the sap of many tree species in geographically distinct regions of the world. Specific substrates associated with this species are largely linked to its ability to assimilate the methanol produced in decaying plant tissues [36].

**Table 1:** Identification, occurrence and fermentation in Durham tube test of yeasts isolated in Amazonian forest reserves.

Yeast species	Sampled medium		Fermentation in Durham tube test
	YNB-D-xylose (n = 40)	YNB-xylan (n = 40)	
**São João da Baliza Forest Reserve**			
*Asterotremella humicola*	2^1^	4	−
*Blastobotrys mokoenaii* 3	1	1	−
*Candida amphixiae* 3,5	−	1	−
*C. boidinii*	5	5	−
*C. gorgasii* 2,3	1	1	−
*C. intermedia*	4	3	−
*C. labiduridarum* 3,4	2	−	−
*C. palmioleophila* 3,5	−	1	−
*C. pseudointermedia* 3,5	−	1	−
*C. quercitrusa*	1	−	−
*C. tropicalis*	1	3	−
*Candida* sp. 2^2,3^	2	1	−
*Candida* sp. 3^2,3,5^	−	2	−
*Cryptococcus diffluens* 3,5	−	1	−
*Cr. laurentii* 5	−	1	−
*Debaryomyces hansenii*	5	2	−
*Kodamaea ohmeri* 3,5	−	1	−
*Lindnera saturnus*	5	1	−
*Meyerozyma guilliermondii*	−	1	−
*Naumavazyma castelli* 3,4	1	−	−
*Scheffersomyces stipitis*	1	−	+
*Schwanniomyces polymorphus* 3	6	2	−
*Sc. vanrijiae* 3	2	2	−
*Spathaspora passalidarum* 3,5	−	5	+
*Spathaspora* sp. 1^2,3^	1	1	−
*Spathaspora* sp. 3^2,3,5^	−	1	−
*Trichosporon mycotoxinivorans*	1	2	−
**Mucajaí Forest Reserve**			
*A. humicola*	6	6	−
*Candida amazonensis* 2,3	3	1	−
*C. blattae* 3,4	1	−	−
*C. boidinii*	5	2	−
*C. intermedia*	−	1	−
*C. maltosa* 3	2	2	−
*C. natalensis* 3,5	−	1	−
*C. quercitrusa*	−	1	−
*C. tropicalis*	14	10	−
*Candida* sp. 1^2,3,5^	−	2	−
*Cr. laurentii* 5	−	1	−
*D. hansenii*	2	2	−
*L. saturnus*	1	1	−
*M. guilliermondii*	4	1	−
*S. stipitis*	−	1	+
*Spathaspora* sp. 2^2,3,4^	1	−	−
*T. mycotoxinivorans*	−	3	−

1 Number of samples in which the yeast was isolated.

2 Novel yeast species.

3 Occurrence restricted to one isolation site.

4 Occurrence restricted to YNB-D-xylose medium.

5 Occurrence restricted to YNB-xylan medium.

The species restricted to the São João da Baliza forest reserve included all *Spathaspora passalidarum* isolates and four new species (*Candida* sp. 2, *Candida* sp. 3, *Spathaspora* sp. 1 and *Spathaspora* sp. 3). *Candida amazonensis*, *Candida* sp. 1 and *Spathaspora* sp. 2 were recovered in the Mucajaí forest reserve. Fifteen species were isolated on only one cultivation media, four in YNB-D-xylose medium (*C. blattae, C. labiduridarum, Naumavazyma castelli* and *Spathaspora* sp. 2) and 11 species in YNB-xylan medium (*C. amphixiae*, *C. natalensis, C. palmioleophila, C. pseudointermedia, Candida* sp. 1, *Candida* sp. 3, *Cryptococcus diffluens, Cr. laurentii, Kodamaea ohmeri, S. passalidarum*, and *Spathaspora* sp. 3). Most known yeast species found in YNB-D-xylose or xylan media have previously been linked to terrestrial environments, such as soil, flowers, fruit, rotting wood, beetle guts, and floricolous insects [42]. Suh et al. [8] reported that *C. intermedia, Meyerozyma guilliermondii*, *Lindnera (Williopsis) saturnus*, and *S. stipitis* were associated with wood-ingesting beetles, and Nguyen et al. [14] observed the same for *S. passalidarum*. Bhadra et al. [43] isolated *Cr. laurentii, D. hansenii* and *K. ohmeri* from tree bark samples. *Candida tropicalis* and *C. maltosa* were isolated from rotten fruit by Rao et al. [10]. *Schwanniomyces polymorphus* and *Sc. vanrijiae* were also isolated from several samples collected in the São João da Baliza forest reserve. These metabolically versatile species have been isolated from soil, tree exudates, and ant hills [44]. Some species, namely *D. hansenii, C. intermedia* and *M. guilliermondii*, were isolated in both culture media and from both collection sites. Two isolates of *B. mokoenaii* were obtained from the São João da Baliza site. This species is rare and known previously from only a single strain obtained from soil in South Africa [45]. Du Preez et al. [46] reported that *B. mokoenaii* is thermotolerant and produces an extracellular endo-β-xylanase comparable to that of *Aureobasidium pullulans*.

### Molecular Identification and Phylogenetic Relationships

**Figure 1:**
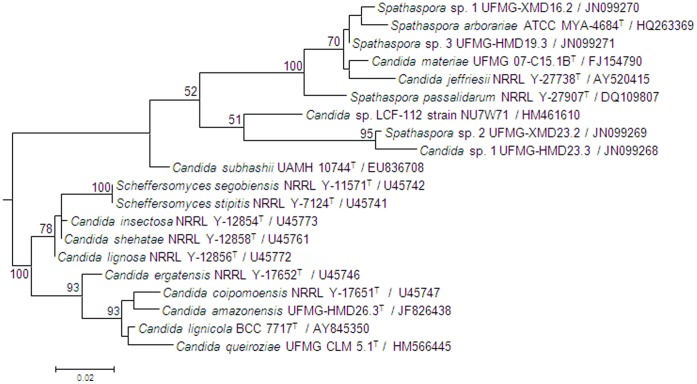
Phylogram of yeast species considered in this study based on the D1/D2 domains of the large subunit ribosomal gene. The maximum likelihood tree was constructed with the Mega5 program following correction of the distances with the Kimura 2-parameter transformation. A total of 499 nucleotide positions were used in the analysis. Bootstrap values of 50% or greater are shown (100 replicates). Bar 0.02 substitutions per nucleotide position.

Some yeast isolates showed three or more non-contiguous nucleotide differences in the D1/D2 domains of the LSU rRNA gene when compared with the most closely related known species, indicating that they might represent novel yeast species. According to Kurtzman et al. [42], isolates of the same species typically have zero to two nucleotide differences in the D1/D2 region of the large subunit of the rRNA gene. Based on this concept, seven new yeast species were found in our studies. Five isolates have been described as a new species named *Candida amazonensis* [47]. The new species belongs to the *Scheffersomyces* clade and differs by nine nucleotide substitutions and six indels in the D1/D2 region of the LSU rRNA gene from *C. coipomoensis*, nine nucleotide substitutions and seven indels from *C. lignicola* and 16 nucleotide substitutions and six indels from *C. queiroziae*, its closest relatives [47]. All yeast species that are phylogenetically related to *C. amazonensis* were also isolated from rotting-wood samples or insects associated with this substrate [36], [48], [49]. The yeast isolates identified as *Candida* sp. 1, *Spathaspora* sp. 1, *Spathaspora* sp. 2 and *Spathaspora* sp. 3 by sequence analysis belong to the *Spathaspora* clade, which contains D-xylose-fermenting yeasts. *Spathaspora* sp. 3 (UFMG-HMD-19.3) and *Spathaspora* sp. 1 (UFMG-XMD-16.2) belong to the same subclade as do *S. arborariae*, *Candida jeffriesii*, and *Candida materiae*. *Spathaspora* sp. 3 (UFMG-HMD-19.3) differs in the D1/D2 domains by eight substitutions from its least divergent relative, *C. materiae*. The smallest degree of sequence divergence observed for *Spathaspora* sp. 1 (UFMG-XMD-16.2) was seven substitutions and four indels from *S. arborariae*. The other two new species, *Spathaspora* sp. 2 (UFMG-XMD-23.2) and *Candida* sp. 1 (UFMG-HMD-23.3), are closely related, forming a separate subclade within the *Spathaspora* clade. They differ by 76 changes in the combined D1/D2 domains (6 substitutions and 5 indels) and ITS region (19 substitutions and 46 indels) and are well separated phylogenetically from other clade members (Fig. 1). The D1/D2 sequences of both isolates of *Spathaspora* sp. 1 (UFMG-XMD-16.2 and UFMG-HMD-16.3) were identical. The same was observed for the isolates of *Candida* sp. 1 (UFMG-HMD-23.3 and UFMG-HMD-25.1). Three isolates were identified as *Candida* sp. 2 (GenBank accession number is JQ695901). This new species belongs to the *Wickerhamomyces* clade and it differs by six nucleotide substitutions from *C. mycetangii*. The later species is associated with tree-boring beetles [36]. *Candida* sp. 3 (GenBank accession number is JQ695900) belongs to the *Lindnera* clade and it differs by six nucleotide substitutions from *Lindnera* (*Pichia) japonica*, a species associated with tree boring insects [50].

Out of the 33 yeast species identified, strains belonging to only two species, *S. passalidarum* and *S. stipitis*, showed gas formation from D-xylose in the Durham tube test. Both species had previously been shown to ferment D-xylose [11], [14]. However, although one new species (*C. amazonensis*) was identified as belonging to the *Scheffersomyces* clade and four as belonging to the *Spathaspora* clade, these isolates exhibited no gas production in the Durham tube test, showing that this test alone is insufficient in screening for D-xylose-fermenting yeasts.

### Fermentation Assays

#### D-xylose culture medium

To evaluate D-xylose fermentation and ethanol production, two strains of *S. stipitis,* six strains of *S. passalidarum,* five strains of *Candida amazonensis* and all strains of the new species belonging to the *Spathaspora* clade were subjected to fermentation assays in D-xylose (50 g/L) culture medium. The results of the fermentation parameters [Y_p/s_^et^ (g/g), ethanol yield; Y_p/s_^xy^ (g/g), xylitol yield; Qp (g/L·h), ethanol productivity; η (%), fermentation efficiency; (%), D-xylose consumption (%)] and cells, ethanol and xylitol concentrations (g/L) are summarized in Table 2. These results were calculated according to the fermentation time (time of maximum ethanol production or time of the end of the fermentation experiment) for each yeast strain.

**Table 2:** Ethanol yield [Y_p/s_^et^ (g/g)], xylitol yield [Y_p/s_^xy^ (g/g)], ethanol productivity [Qp (g/L•h)], fermentation efficiency [η (%)], D-xylose consumption (%), cell concentration (g/L), ethanol concentration (g/L) and xylitol concentration (g/L) in D-xylose culture medium assays.

Yeast species	Yeast strain	D-xylose consumption (%)^1^	Cells (g/L)	Y_p/s_^et^ (g/g)^2^	Qp (g/L•h)^3^	Η (%)^4^	Ethanol concentration(g/L)	Y_p/s_^xy^ (g/g)^5^	Xylitol concentration(g/L)	FermentationTime (h)^6^
*Candida lignosa*	CBS 4705	76.2	7.7	0.40	0.60	79.4	14.5	0.03	1.0	24
*Scheffersomyces stipitis*	NRRL Y-7124	89.0	10.9	0.35	0.62	69.5	15.0	0.02	1.3	24
*S. stipitis*	UFMG-XMD-15.2	84.5	12.9	0.28	0.51	55.2	12.3	0.04	1.9	24
*S. stipitis*	UFMG-HMD-32.1	98.8	14.2	0.22	0.23	44.1	11.1	–	–	48
*C. amazonensis* sp. nov.	UFMG-XMD-24.1	99.9	10.6	0.08	0.08	15.3	4.0	0.59	25.2	48
*C. amazonensis* sp. nov.	UFMG-XMD-26.2	99.8	10.7	0.08	0.08	14.6	4.0	0.58	25.2	48
*C. amazonensis* sp. nov.	UFMG-HMD-26.3	99.9	10.7	0.07	0.08	13.4	3.7	0.57	24.8	48
*C. amazonensis* sp. nov.	UFMG-XMD-40.2	99.9	11.8	0.07	0.08	14.4	3.8	0.55	23.7	48
*C. amazonensis* sp. nov.	UFMG-XMD-40.3	99.9	11.1	0.07	0.08	14.0	3.7	0.56	23.9	48
*Spathaspora passalidarum*	UFMG-HMD-1.1	98.4	8.7	0.36	0.75	70.0	18.0	0.03	1.5	24
*S. passalidarum*	UFMG-HMD-1.3	98.1	9.8	0.35	0.72	68.4	17.2	0.04	2.2	24
*S.passalidarum*	UFMG-HMD-2.1	98.4	10.7	0.31	0.62	61.0	15.0	0.02	1.1	24
*S. passalidarum*	UFMG-HMD-10.2	98.4	10.6	0.33	0.69	65.4	16.6	0.02	1.2	24
*S. passalidarum*	UFMG-HMD-14.1	98.3	10.2	0.37	0.68	71.6	16.4	0.02	1.1	24
*S. passalidarum*	UFMG-HMD-16.2	98.3	9.9	0.33	0.64	64.2	15.3	0.02	1.0	24
*Spathaspora* sp. 1	UFMG-XMD-16.2	86.8	7.4	0.33	0.27	65.4	13.1	0.21	7.8	48
*Spathaspora* sp. 1	UFMG-HMD-16.3	84.0	6.3	0.27	0.22	53.8	10.7	0.17	6.7	48
*Spathaspora* sp. 2	UFMG-XMD-23.2	89.8	7.4	0.26	0.21	50.8	10.2	0.19	7.0	48
*Spathaspora* sp. 3	UFMG-HMD-19.3	55.2	5.6	0.13	0.07	26.2	3.3	0.16	3.9	48
*Candida* sp 1	UFMG-HMD-23.3	60.2	9.3	0.18	0.10	35.8	4.9	0.13	3.3	48
Candida sp 1	UFMG-HMD-25.1	52.3	9.0	0.14	0.06	27.0	3.1	0.22	4.6	48

1 D-xylose consumption (%) – percentage of initial D-xylose consumed.

2 Y_p/s_^et^ (g/g) – ethanol yield: correlation between ethanol (ΔP_ethanol_) produced and D-xylose (ΔS_xylose_) consumed.

3 Qp (g/L•h) – ethanol productivity: ratio of ethanol concentration (g/L) and fermentation time (h).

4 η (%) – fermentation efficiency: percentage of the maximum theoretical ethanol yield (0.51 g ethanol/g D-xylose).

5 Y_p/s_^xy^ (g/g) – xylitol yield: correlation between xylitol (ΔP_xylitol_) produced and D-xylose (ΔS_xylose_) consumed.

6 Time when the maximum ethanol production (g/L) value was attained or time of the end of the fermentation experiment.

Fermentation results revealed that all yeasts tested were able to consume D-xylose, with the consumption rates ranging from 52.3% to practically 100% in 48 h. Da Cunha-Pereira et al. [15] found an efficiency of 84% for D-xylose utilization in 96 h by *S. arborariae* UFMG-HM 19.1A in synthetic medium containing initial concentrations of 20 g/L xylose, 20 g/L glucose and 10 g/L arabinose. Agbogbo et al. [51] reported 100% D-xylose consumption after 120 h by *S. stipitis* CBS 6054 in synthetic medium formulated with 60 g/L D-xylose. Agbogbo and Wenger [52] demonstrated a D-xylose consumption over 85% in 48 h by *S. stipitis* CBS 6054 in synthetic medium with approximately 21 g/L D-xylose and 5.8 g/L glucose. According to du Preez and Prior [53], *C. shehatae* CBS 2779 consumed 100% xylose in 28 h from medium containing 20 g/L xylose. Among the microorganisms studied in our work, the isolates of *C. amazonensis* showed the highest D-xylose consumption rates, approaching 100% of consumption (values ranged from 99.8% to 99.9%) in 48 h, whereas *S. passalidarum* showed the fastest D-xylose consumption rate, 98%, in 24 h. Among the new species, both *Spathaspora* sp. 1 and *Spathaspora* sp. 2 consumed up to 80% of D-xylose in 48 h, while *Spathaspora* sp. 3 and *Candida* sp. 1 consumed 52.3% to 60.2% of D-xylose, respectively, in 48 h. Strains of *S. stipitis* achieved similar maximum D-xylose consumption values, approximately 98.8%, in 48 h. These results show, in general, high and fast D-xylose consumption by most of the yeasts studied in this work. Furthermore, we also observed that a specifically high and fast D-xylose consumption behavior can likely be associated with *C. amazonensis*, *S. passalidarum* and *S. stipitis* strains isolated in this work, but not with all species from the genus *Spathaspora*.

Ethanol production was observed during the fermentation assay, confirming the ability to ferment D-xylose to ethanol by the microorganisms tested. The highest ethanol concentration values were produced by the *S. passalidarum* strains (a species that exhibited gas production in the Durham tube test), ranging from 15 g/L to 18 g/L in 24 h. *Candida amazonensis*, *Spathaspora* sp. 3 and *Candida* sp. 1 were at the other end of the spectrum, showing the lowest ethanol concentration values, between 3.1 g/L and 4.9 g/L in 48 h. *Scheffersomyces stipitis* UFMG-XMD-15.2 and UFMG-HMD-32.1, that exhibited gas production in the Durham tube test, produced ethanol with similar concentrations, 12.3 g/L and 11.1 g/L, respectively, but at different fermentation times, 24 h and 48 h, respectively. The variations observed can probably be associated with physiological differences between strains of the same species. Ethanol production of 15 g/L was observed by the yeast *S. arborariae* UFMG-HM-19.1A in approximately 72 h from 20 g/L xylose, 20 g/L glucose and 10 g/L arabinose [15]. *Candida shehatae* CBS 2779 reached 13.1 g/L ethanol in 28 h in synthetic medium containing 20 g/L xylose [53]. *Scheffersomyces stipitis* CBS 6054 was able to produce 24.3 g/L ethanol in 120 h in medium with 60 g/L D-xylose [51]. Agbogbo and Wenger [53] also showed the production of approximately 8.7 g/L ethanol in 48 h by *S. stipitis* CBS 6054 from 20 g/L xylose. In general, *S. passalidarum* strains showed the best potential for ethanol production in the conditions tested because they produced the highest ethanol concentrations in a short period of time.

*Candida lignosa* CBS 4705 (positive control) presented the highest ethanol yield (Y_p/s_^et^  = 0.4 g/g) and fermentation efficiency (η = 79.4%). *S. passalidarum* strains showed the highest ethanol productivity (Qp = 0.62 g/L·h to 0.75 g/L·h), with minimum and maximum Y_p/s_^et^ values between 0.31 g/g and 0.37 g/g and η between 61% and 71.6%. The new species *Spathaspora* sp. 1 UFMG-XMD-16.2, *Spathaspora* sp. 1 UFMG-HMD-16.3 and *Spathaspora* sp. 2 UFMG-XMD-23.2 showed better ethanol fermentation results than *C. amazonensis* strains, *Spathaspora* sp. 3, *Candida* sp. 1 UFMG-HMD-23.3 and *Candida* sp. 1 UFMG-HMD-25.1 (Table 2). Da Cunha-Pereira et al. [15] found a Y_p/s_^et^ and Qp equivalent to 0.45 g/g and 0.21 g/L·h, respectively, for *S. arborariae* UFMG-HM-19.1A. *Candida shehatae* CBS 2779 and *S. stipitis* CBS 6054 showed Y_p/s_^et^ values equal to 0.37 g/g and 0.44 g/g, respectively, and Qp equivalent to 0.47 g/L·h and 0.20 g/L·h, respectively [51], [53]. Overall, *S. passalidarum* strains showed promising results for ethanol production under the conditions tested, considering their remarkably high ethanol productivity (Qp = 0.62 g/L·h to 0.75 g/L·h) and fermentation efficiency of approximately 70% (η = 61% to 71.6%), in 24 h. However, Y_p/s_^et^ values found in *S. passalidarum* strains (0.31 g/g and 0.37 g/g) were lower than those found in *S. arborariae* UFMG-HM-19.1A and *S. stipitis* CBS 6054, but it must be taken into consideration the different experimental conditions employed for these yeasts. *Spathaspora arborariae* UFMG-HM-19.1A produced Y_p/s_^et^ equal to 0.45 g/g in medium containing 20 g/L xylose, but also 20 g/L glucose and 10 g/L arabinose, and its maximum ethanol production was achieved in approximately 72 h [15], while *S. stipitis* CBS 6054 showed Y_p/s_^et^ equal to 0.44 g/g in medium containing 60 g/L xylose and took 120 h to reach its maximum ethanol concentration [51]. In our study, D-xylose (50 g/L) was used as the sole carbon source, and *S. passalidarum* strains reached maximum ethanol production in 24 h.

A good understanding of ethanol production from xylose requires a knowledge of both the experimental conditions and the enzymes responsible for xylose metabolism. In yeasts, fermentation of xylose to ethanol is strongly dependent on NADH-linked xylose reductase (XR). In *Scheffersomyces stipitis*, XR preferentially uses NADPH, although it can also use NADH [54]. Recently, it was shown that in *S. passalidarum* xylose is converted by means of an NADH-preferring XR [16]. These observations are compatible with the higher ethanol yields achieved by strains of *S. passalidarum* and *S. stipitis* tested in this work. The impact of XR activities and their co-factor preferences in on ethanol production among these new xylose-fermenting yeasts will be the focus of future work.

Yeast strains that achieved their maximum ethanol concentration in 24 h were observed to undergo a decrease in ethanol concentration after this period (data not shown). This decrease probably results from ethanol assimilation by the yeasts as a consequence of the rapid depletion of sugar from the medium while oxygen remains available. This behavior has previously been reported for yeasts in studies utilizing both synthetic medium and/or lignocellulosic hydrolysates [9], [12], [15], [52], [55].

Although ethanol was the main product obtained in these fermentation assays, by-products such as xylitol and glycerol were also found. Glycerol was present at low concentrations (on average ≤0.05 g/L, maximum value of 1.4 g/L, data not shown), whereas xylitol was produced at higher concentrations. The lowest xylitol values (0 g/L to 2.2 g/L) were obtained for *S. stipitis, C. lignosa* and *S. passalidarum.* Ferreira et al. [12] suggested that the formation of low amounts of by-product is important characteristics of the yeast strain, as they allow elevated formation of the main fermentation product, ethanol, to be obtained. The new *Spathaspora* species that was identified in this study produced xylitol concentrations between 3.3 g/L and 7.8 g/L in 48 h (Y_p/s_^xy^ between 0.13 g/g and 0.22 g/g) and, together with *C. amazonensis*, produced lower ethanol concentrations than the other species tested. Another species from *Spathaspora* clade, *S. arborariae* UFMG-HMD-19.1A has already been shown to produce xylitol, at 5 g/L in 108 h in synthetic medium [15]. *Candida amazonensis* was responsible for the highest xylitol production values found in this assay (26.1 g/L to 27.8 g/L in 24 h, Y_p/s_^xi^ between 0.62 g/g and 0.67 g/g, data not shown). These values were much higher than those found for ethanol production by all yeasts tested.

After 24 h, a decrease in the xylitol concentration occurred, indicating that this product was being used as carbon source, presumably following D-xylose depletion and also due the oxygen availability in the medium. Xylitol is one of the most expensive polyol sweeteners in the world market and has been the subject of specific health claims. It is suitable for diabetics and is recommended for oral health and parenteral nutrition [56]. The biotechnological production of xylitol using microorganisms such as yeasts and fungi is of economic interest and presents some advantages when compared with conventional xylitol production by the chemical reduction of D-xylose, as this latter process is characterized by high costs due to difficulties in separation and purification steps [39], [57], [58]. Thus, *C. amazonensis* isolates obtained in this work can be studied in the future for direct xylitol production from D-xylose in hemicellulosic hydrolysates. Further studies are needed to establish the best cultivation conditions for this process.

#### Sugarcane bagasse hemicellulosic hydrolysate

After performing D-xylose fermentation assays, we selected a number of strains for fermentation tests in sugarcane bagasse hydrolysate. We picked the strains or species that presented the highest rates of D-xylose consumption and ethanol yields (Y_p/s_^et^), specifically *S. passalidarum* UFMG-HMD-1.1 and UFMG-HMD-14.1, *S. stipitis* UFMG-XMD-15.2, *Spathaspora* sp. 1 UFMG-XMD-16.2 and *Spathaspora* sp. 2 UFMG-XMD-23.2. *Candida amazonensis*, *Spathaspora* sp. 3 and *Candida* sp. 1 were not selected due to their low ethanol production in D-xylose culture medium.

The fermentation parameter results [Y_p/s_^et^ (g/g), ethanol yield; Y_p/s_^xy^ (g/g), xylitol yield; Qp (g/L·h), ethanol productivity; η (%), fermentation efficiency; sugar (D-xylose and glucose) consumption (%)] and cells, ethanol and xylitol concentrations (g/L) in sugarcane bagasse hemicellulosic hydrolysates are summarized in Table 3. These results were also calculated based on the fermentation time (time of maximum ethanol production or time of the end of the fermentation experiment) for each yeast strain.

**Table 3:** Ethanol yield [Y_p/s_^et^ (g/g)], xylitol yield [Y_p/s_^xy^ (g/g)], ethanol productivity [Qp (g/L•h)], fermentation efficiency [η (%)], sugar (D-xylose and glucose) consumption (%), cell concentration (g/L), ethanol concentration (g/L) and xylitol concentration (g/L) in sugarcane bagasse hemicellulosic hydrolysate assays.

Yeast species	Yeast strains	Sugars consumption (%)^1^	Cells (g/L)	Y_p/s_^et^ (g/g)^2^	Qp (g/L•h)^3^	Η (%)^4^	Ethanol concentration (g/L)	Y_p/s_^xy^ (g/g)^5^	Xylitol concentration (g/L)	Fermentation time (h)^6^
*Candida lignosa*	CBS 4705	55.3	7.8	0.16	0.05	31.6	4.6	–	–	96
*Scheffersomyces stipitis*	NRRL Y-7124	34.9	3.4	0.25	0.10	49.3	4.9	–	–	48
*S. stipitis*	UFMG-XMD-15.2	80.9	12.7	0.34	0.20	65.7	14.1	–	–	72
*Spathaspora passalidarum*	UFMG-HMD-1.1	84.9	13.4	0.20	0.09	40.0	8.8	–	–	96
*S. passalidarum*	UFMG-HMD-14.1	91.0	13.1	0.18	0.10	36.0	9.5	–	–	96
*Spathaspora* sp. 1	UFMG-XMD-16.2	72.9	7.9	0.23	0.10	46.0	9.3	0.57	18.2	96
*Spathaspora* sp. 2	UFMG-XMD-23.2	68.6	6.4	0.22	0.08	42.4	7.2	0.61	17.1	96

1 Sugar consumption (%) – percentage of the initial D-xylose and glucose consumed.

2 Y_p/s_^et^ (g/g) – ethanol yield: correlation between ethanol (ΔP_ethanol_) produced and D-xylose and glucose (ΔS_sugars_) consumed.

3 Y_p/s_^xy^ (g/g) – xylitol yield: correlation between xylitol (ΔP_xylitol_) produced and D-xylose (ΔS_xylose_) consumed.

4 Qp (g/L·h) – ethanol productivity: ratio of ethanol concentration (g/L) and fermentation time (h).

5 η (%) – fermentation efficiency: percentage of the maximum theoretical ethanol yield (0.51 g ethanol/g D-xylose and glucose).

6 Time when the maximum ethanol production (g/L) value was attained or time of the end of the fermentation experiment.

Taking only into account the glucose and D-xylose present in the hydrolysates, *S. stipitis* UFMG-XMD-15.2 showed the highest sugar consumption, equal to 93.4% in 96 h (data not shown), followed by *S. passalidarum* strains UFMG-HMD-1.1 (84.9%) and UFMG-HMD-14.1 (91%) in 96 h. *Spathaspora* sp. 2 UFMG-XMD-23.2 and *Spathaspora* sp. 1 UFMG-XMD-16.2 presented values of 68.6% and 72.9%, respectively, whereas *C. lignosa* CBS 4705 (positive control) showed the lowest consumption value, equal to 55.3% in 96 h. *Scheffersomyces stipitis* NRRL Y-7124 (positive control) consumed 69.8% of glucose and D-xylose (data not shown) in 96 h, similar to the amount consumed by *Spathaspora* sp. 2. Da Cunha-Pereira et al. [15] observed that *S. arborariae* UFMG-HM-19.1A consumed glucose and xylose at 100% and 45% in approximately 120 h and 240 h, respectively, in rice hull hydrolysate containing 35 g/L glucose, 13 g/L xylose and 4 g/L arabinose. *Scheffersomyces stipitis* UFMG-IMH-43.2 consumed, on average, 83.6% of glucose and xylose in 192 h, when cultivated in sugarcane bagasse hydrolysate with an initial concentration of 52.5 g/L xylose [12]. Chandel et al. [59] reported a total sugar consumption of 85.9% after 24 h fermentation by *C. shehatae* NCIM 3501 in sugarcane bagasse hydrolysate with 20 g/L total sugars. Among the yeasts tested in our assays, glucose and D-xylose were initially metabolized at the same time, but the glucose consumption rate was clearly faster than that of D-xylose. After glucose exhaustion, D-xylose was consumed much faster, and this behavior was observed after 12 h by *S. passalidarum* strains and *S. stipitis* UFMG-XMD-15.2, after 24 h by *S. stipitis* NRRL-Y 7124 and *C. lignosa* CBS 4705 and after 48 h by *Spathaspora* sp. 1 and *Spathaspora* sp. 2 (data not shown). When compared with the results obtained in other studies on hydrolysate fermentation, *S. stipitis* UFMG-XMD-15.2 and the *S. passalidarum* strains tested in our work showed excellent D-xylose and glucose consumption results, when the initial D-xylose (50.2 g/L) and glucose (5.3 g/L) concentrations and the time of consumption (96 h) of the majority (84.9% and 93.4%) of these sugars are considered. Unlike D-xylose and glucose, L-arabinose consumption was not observed by the yeasts during the fermentation time studied. The absence of L-arabinose consumption was also observed in fermentations carried out with *S. stipitis* UFMG-IMH-43.2 in experiments similar to those performed in this work [12].

Ethanol production was observed within 12 h of cultivation by the yeasts tested. *Scheffersomyces stipitis* NRRL Y-7124 produced a maximum concentration of 4.9 g/L ethanol within 48 h of fermentation. *Candida lignosa* CBS 4705, *S. passalidarum* UFMG-HMD-1.1 and UFMG-HMD-14.1 reached maximum ethanol concentrations of 4.6 g/L, 8.8 g/L and 9.5 g/L, respectively, in 96 h. *S. stipitis* UFMG-XMD-15.2 was responsible for the highest ethanol production, corresponding to 14.1 g/L in 72 h. An ethanol production greater than 15 g/L was observed for *S. arborariae* UFMG-HM-19.1A after 50 h in rice hull hydrolysate [15]. *Candida shehatae* NCIM 3501 produced 5.2 g/L ethanol after 24 h in sugarcane bagasse hydrolysate [59]. *Spathaspora passalidarum* UFMG-HMD-1.1 and UFMG-HMD-14.1 showed ethanol concentration results similar to that reported by *S. stipitis* UFMG-IMH-43.2, which presented an ethanol production equal to 9.1 g/L, but in 192 h [12]. Still, when the strains tested in this study were compared with *S. stipitis* UFMG-IMH-43.2, *S. stipitis* UFMG-XMD-15.2 produced a higher ethanol concentration (14.1 g/L) at a much faster rate (72 h). It must be noted that both studies used sugarcane bagasse hemicellulosic hydrolysate with similar experimental conditions (including the initial concentration of D-xylose, supplementation, agitation and incubation temperature). Considering the initial high D-xylose concentration (50.2 g/L) relative to the glucose concentration (5.3 g/L) and the remarkably high ethanol production by *S. stipitis* UFMG-XMD-15.2 relative to other species tested here, the hydrolysate fermentation study results suggest that this strain is a promising tool for producing ethanol from D-xylose.

*Scheffersomyces stipitis* UFMG-XMD-15.2 presented the highest ethanol yield (Y_p/s_^et^  = 0.34 g/g), ethanol productivity (Qp = 0.2 g.L/h) and fermentation efficiency (η = 65.7%). *Spathaspora passalidarum* strains UFMG-HMD-1.1 and UFMG-HMD-14.1 showed Y_p/s_^et^ values between 0.18 g/g and 0.2 g/g, ethanol productivity between 0.09 and 0.1 g/L·h and η between 36% and 40%, whereas *Spathaspora* sp. 2 and *Spathaspora* sp. 1 presented ethanol yields between 0.22 g/g and 0.23 g/g, Qp between 0.08 g/L·h and 0.1 g/L·h and η between 42.4% and 46%. Da Cunha-Pereira et al. [15] found a Y_p/s_^et^ and Qp equivalent to 0.45 g/g and 0.16 g/L·h, respectively, for *S. arborariae* UFMG-HM-19.1A in a fermentation assay using rice hull hydrolysate. *Candida shehatae* NCIM 3501 showed Y_p/s_^et^ equal to 0.3 g/g and Qp equivalent to 0.22 g/L·h in sugarcane bagasse hydrolysate [59], results similar to those found for *S. stipitis* UFMG-XMD-15.2. Ferreira et al. [12] found Y_p/s_^et^ and Qp equal to 0.17 g/g and 0.05 g/L·h, respectively, for *S. stipitis* UFMG-IMH-43.2, showing that ethanol fermentation parameters presented by the strains isolated in our study were better than those observed for *S. stipitis* UFMG-IMH-43.2. When compared with the ethanol yield of *S. arborariae* [15], the yeast strains isolated in this study showed lower Y_p/s_^et^ in hemicellulosic hydrolysate. *Spathaspora arborariae* UFMG-HM-19.1A produced Y_p/s_^et^ equal to 0.45 g/g in rice hull hydrolysate containing glucose as the main sugar (35 g/L), with an initial xylose concentration (13 g/L) that was much lower than that employed in our work (50.2 g/L). Despite the higher Y_p/s_^et^ presented by *S. arborariae* in rice hull hydrolysates, *S. stipitis* UFMG-XMD-15.2 showed a Qp (0.2 g/L·h) higher than that observed for *S. arborariae* (0.16 g/L·h) in our experiments.

Ethanol consumption was observed by *S. stipitis* NRRL Y-7124 after 48 h and *S. stipitis* UFMG-XMD-15.2 after 72 h (time of maximum ethanol production). Glycerol was found at low concentrations in *S. passalidarum* UFMG-HMD-1.1 (0.05 g/L), *S. passalidarum* UFMG-HMD-14.1 (0.1 g/L), *S. stipitis* UFMG-XMD-15.2 (0.7 g/L), *Spathaspora* sp. 1 UFMG-XMD-16.2 (0.2 g/L) and *Spathaspora* sp. 2 UFMG-XMD-23.2 (0.6 g/L). Except for the new species identified, xylitol production was not observed for the other yeasts studied. Again, as suggested by Ferreira et al. [12], the low production rates of xylitol and glycerol may be important characteristics of the yeast strain that should allow higher production of the main fermentation product, ethanol, by the microorganism, a physiological characteristic desirable in our study.

*Spathaspora* sp. 1 UFMG-XMD-15.2 and *Spathaspora* sp. 2 UFMG-XMD-23.2 were the only yeasts that were observed to produce xylitol (18.2 g/L and 17.1 g/L, respectively) in this assay (Table 3). These yeasts produced markedly higher xylitol concentrations in hydrolysate than in D-xylose culture medium. Xylitol yield values that were found for these new species were considerably higher than that found for a previously identified strain of *C. tropicalis* from the soil (Y_p/s_^xy^  = 0.45 g/g), tested in a fermentation assay on sugarcane hydrolysate with an initial xylose concentration of 30 g/L [39]. The xylitol production (g/L) and Y_p/s_^xy^ results found in our work were also higher than those reported by Silva and Roberto [60] for *C. gulliermondii* FTI 20037. These authors observed a xylitol production of 17 g/L and a Y_p/s_^xy^ of 0.35 g/g after 120 h for *C. gulliermondii* FTI 20037 in a rice straw hemicellulosic hydrolysate fermentation assay, with an initial xylose concentration of 90 g/L. The biotechnological production of xylitol from crude hemicellulosic hydrolysates could be a valuable economic alternative to the expensive chemical production of xylitol from D-xylose [39], [61]. Our results for xylitol production by *Spathaspora* sp. 1 and *Spathaspora* sp. 2 are promising and warrant future testing of the production of this polyol from xylose fermentation in hemicellulosic hydrolysates.

Our study investigated the ability of yeasts to produce ethanol and/or xylitol while consuming D-xylose in D-xylose culture medium and produce ethanol and/or xylitol while consuming D-xylose and glucose in sugarcane bagasse hemicellulosic hydrolysate. *S. stipitis* UFMG-XMD-15.2 is of particular interest because it efficiently converted D-xylose to ethanol and grew well in sugarcane bagasse hemicellulosic hydrolysate. *Spathaspora passalidarum* strains were also observed to grow in hydrolysates and produce ethanol. *Candida amazonensis* was notable for its potential use in the biotechnological production of xylitol. The newly identified yeast strains *Spathaspora* sp. 1 and *Spathaspora* sp. 2 were similarly found to potentially produce xylitol in hemicellulosic hydrolysate. Future studies are needed to test their production of this polyol. As this work is the first to use these yeasts in fermentation assays, further studies are required to optimize cultivation conditions (nutritional dependence, initial substrate concentration, initial cell concentration, pH, temperature and aeration) for the efficient conversion of D-xylose into ethanol or xylitol.

## Supporting Information

Table S1 **List of the new yeast species isolated in this work and their respective GenBank deposit numbers.**
(DOC)
