# Supplementary material for: Diversity and Physiological Characterization of D-Xylose-Fermenting Yeasts Isolated from the Brazilian Amazonian Forest
Source: PLoS One. 2012 Aug 13;7(8):e43135. doi: 10.1371/journal.pone.0043135 (PMC3418277; doi:10.1371/journal.pone.0043135)
Supplement: Table S1 — List of the new yeast species isolated in this work and their respective GenBank deposit numbers. (DOC) [file pone.0043135.s001.doc]

Table S1 - List of the new yeast species isolated in this work and their respective GenBank deposit numbers.

| Yeast species/Strain number | GenBank deposit number |
| --- | --- |
| *Spathaspora* sp*.*3 HMD19.3 | JN099271 |
| *Candida amazonensis* UFMG-HMD26.3 | JF826438 |
| *Spathaspora* sp. 1 UFMG-XMD16.2 | JN099270 |
| *Candida* sp. 1 UFMG-HMD23.3 | JN099268 |
| *Spathaspora* sp. 2 UFMG-XMD23.2 | JN099269 |
| *Candida* sp. 2 UFMG-XMD-16.4 | JQ695901 |
| *Candida* sp. 3 UFMG-HMD-7.2 | JQ695900 |
